# Is endoscopy beneficial in pediatric laparoscopic gastrostomy insertion; A 9-year comparative study

**DOI:** 10.3389/fped.2022.950867

**Published:** 2022-08-11

**Authors:** Rana Bitar, Ajia Syed, Amer Azaz, David Rawat, Mohamed Hobeldin, Mohamad Miqdady, Seifeleslam Abdelsalam

**Affiliations:** Sheikh Khalifa Medical City, Abu Dhabi, United Arab Emirates

**Keywords:** gastrostomy, percutaneous endoscopic gastrostomy, laparoscopic-assisted gastrostomy, laparoscopic-assisted percutaneous endoscopic gastrostomy, pediatric

## Abstract

**Objectives:**

Advancements in pediatric percutaneous endoscopic gastrostomy placement (PEG), laparoscopic-assisted gastrostomy (LAG) technique, and laparoscopic-assisted percutaneous endoscopic gastrostomy (LAPEG) procedure have opened up new options for gastrostomy tube placement. LAPEG utilizes endoscopy and laparoscopy for gastrostomy insertion. This review compares the outcomes and complications of LAG and LAPEG techniques in children.

**Methods:**

All LAG and LAPEG gastrostomy tube placements in children from September 2010 to September 2019 were reviewed retrospectively. Patient demographic, along with procedural and 1-year complication data, were collected.

**Results:**

In total, 92/181 of gastrostomies were LAG and 89/181 were LAPEG. The mean age, weight and patient characteristics were comparable. Conversion rate was 1% in both groups (*p* = 0.74), there was no peritoneal leak in either group, a minor serosal injury to the stomach was seen in 1 patient in LAG with no bowel injury in LAPEG cohort (*p* = 0.51), need for re-operation was 1 and 2% in LAG and LAPEG, respectively (*p* = 0.49), early tube dislodgement was in 8 (9%) patients in LAG and 7 (6%) in LAPEG (*p* = 0.53) and wound infection was 13/92 in LAG and 11/89 in LAPEG (*p* = 0.8). The median operative time for LAPEG was less than LAG (*p* < 0.001) by 11 min but the median length of hospital stay was not significantly different (*p* < 0.096).

**Conclusion:**

Both LAG and LAPEG techniques in children are safe with comparable complication rates and length of hospital stay, the addition of endoscopy to LAG allowed for shorter operative time in the LAPEG technique.

## What is known

- Laparoscopic-assisted percutaneous endoscopic gastrostomy (LAPEG) was introduced in 1993 as a gastrostomy placement technique that utilizes both endoscopy and laparoscopy.
- LAPEG can be used when percutaneous endoscopic gastrostomy (PEG) is contraindicated or carries increased complication risk.
- Case reports and case series have demonstrated the high safety profile of LAPEG.
- There is only one comparative study comparing laparoscopic-assisted gastrostomy (LAG) to LAPEG in adults (1).

## What is new

- This is the first study comparing LAPEG and LAG in children.
- LAPEG and LAG are both safe with minimal complication rate.
- The addition of endoscopy in LAG makes the procedure shorter.

## Introduction

Gastrostomy tube insertion is a routinely performed procedure in children who require long-term enteral feeding support (1). Gastrostomy is performed in children with underlying oncological disorders, metabolic, renal disorders, neurological pathologies, and congenital or acquired gastrointestinal tract conditions in which oral intake is hindered. Several technical approaches are used for gastrostomy insertion, including percutaneous endoscopic gastrostomy (PEG), percutaneous image guided gastrostomy (PIG), laparoscopic-assisted gastrostomy (LAG), and laparoscopic-assisted percutaneous endoscopic gastrostomy (LAPEG) (2). LAPEG, the most recently introduced technique, simultaneously utilizes both laparoscopic and endoscopic guidance for better visualization during the procedure (3). Typically, one gastrostomy insertion approach is favored over another based on the clinical scenario, facilities, and the clinician’s expertise (4).

Our institution has traditionally used open techniques for gastrostomy placement and very few patients received percutaneous endoscopic gastrostomies due to our concern about risk of possible organ injury associated with PEG. Gastrostomies were inserted for patients who required prolonged tube enteral nutrition (EN) secondary to gastrointestinal and surgical bowel abnormalities, neurological disorders, syndromic disease, increased nutritional requirement from renal, cardiac, and respiratory disease and for metabolic patients who require specialized feeding. Over the past 10 years, we have shifted our clinical practice to lesser invasive techniques, including LAG and LAPEG. Although recent studies have sought to compare outcomes between different modalities and confirmed the safety of laparoscopic techniques (5), the benefit of incorporating endoscopy with laparoscopy, as seen in LAPEG, has not been previously evaluated in children. Therefore, this study aims to compare outcomes and major complications between LAG and LAPEG in the pediatric population.

## Materials and methods

We performed a retrospective analysis of the LAG and LAPEG procedures performed on children over a 9-year period from September 2010 to September 2019 at the Sheikh Khalifa Medical City, the main tertiary-care pediatric hospital in the United Arab Emirates. Approval was obtained from the Institutional Review Board for Research and Ethics Committee. All children under the age of 16 who underwent LAG or LAPEG tube placement during the study period were included in the study. Patients who had an open gastrostomy, percutaneous interventional radiologic gastrostomy (PIRG), and gastrostomy insertion with fundoplication were not included in the study. The minimum follow-up was 1 year. All the procedures were performed after obtaining informed consent from the patients’ parents or legal guardians. The decision for LAPEG and LAG insertion was based on primary physician specialty referral. If the primary physician referred the patient to pediatric gastroenterology, then the patient underwent LAPEG, however, if the patient was referred to the pediatric surgeons, then the patient would undergo LAG. Factors such as obesity, patient weight and previous abdominal surgery did in influence the choice of gastrostomy insertion technique. We have four pediatric gastroenterologists and four pediatric surgeons within our institution and they all were involved in the gastrostomy placement.

A list of all patients undergoing LAG and LAPEG under 16 years was obtained from the operating room electronic record. Data including patient demographics (age, gender, and weight), significant past medical history for cardiac disease or abdominal surgery, pre-operative location of the patient in Pediatric Intensive Care Unit (PICU) or general ward and American Society of Anesthesia (ASA) score were collected from the electronic medical patient records. The primary outcome points were success of the procedure, operative time, length of stay (LOS), rate of surgical site infection, procedure-related complications, and mortality rate. The operative time was calculated only for the patients who underwent isolated gastrostomy insertion, children who underwent another surgical procedure at the time of gastrostomy insertion were excluded when calculating the operative time. Children were discharged when they were deemed clinically stable and after they had achieved the feeding goal set by the pediatric dietician as per the patient needs. Only elective LAG and LAPEG cases were included in the LOS analysis, elective cases included the cases who were planned for gastrostomy insertion through clinic and were not chronic inpatients at the time of the gastrostomy insertion. Patients who were in hospital for prolonged medical or surgical management unrelated to gastrostomy care were excluded. Procedure complications were sub-grouped into intraoperative and postoperative. Intraoperative complications included adjacent bowel injury, intraperitoneal leak and conversion from the planned technique to an alternative gastrostomy placement method. Postoperative complications included re-intervention under general anesthesia, surgical site infection, and early tube dislodgement. Re-intervention under anesthesia was defined as any procedure undertaken under general anesthesia that was performed in relation to a gastrostomy related complication within the first 6 weeks of insertion. Surgical site infection was identified as erythema, induration, exudate, or purulent secretion at the surgical gastrostomy site within 30 days of the procedure (6). A wound culture was obtained any time considered necessary and treatment with antibiotics was prescribed when considered indicated, which was at the surgeon or gastroenterologists’ discretion. Early tube dislodgement was characterized as dislodgement of the feeding tube within 6 weeks of insertion.

The continuous variables are expressed as mean and standard deviations, whereas all the categorical variables are calculated as frequencies and percentages. Median operative time and length of hospital stay were calculated for both procedures because the data were not normally distributed. Statistical analyses were performed utilizing Fisher’s exact test for categorical variables and Student’s *t*-test for all continuous variables. A *p*-value of <0.05 was considered statistically significant. Statistical analysis was performed using IBM SPSS version 22.0 for Windows.

## Operative technique

### Laparoscopic-assisted percutaneous endoscopic gastrostomy

All LAPEG procedures were performed under general anesthesia. We used the push technique to insert a balloon gastrostomy device with the aid of laparoscopy. Since the procedure required the deployment of both endoscopic and laparoscopic techniques, both a pediatric gastroenterologist and a pediatric surgeon were involved.

**FIGURE 1:**
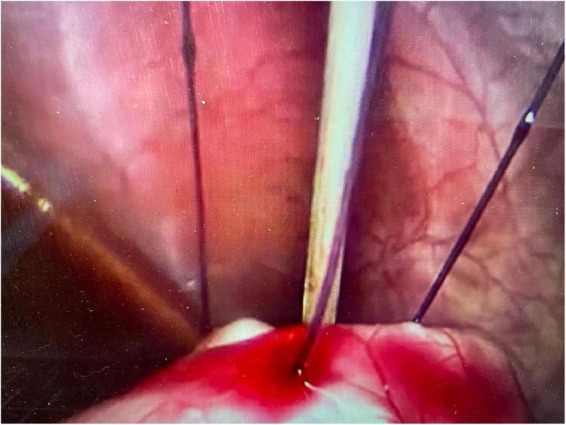
The stomach fixed to the abdominal wall under laparoscopic visualisation.

**FIGURE 2:**
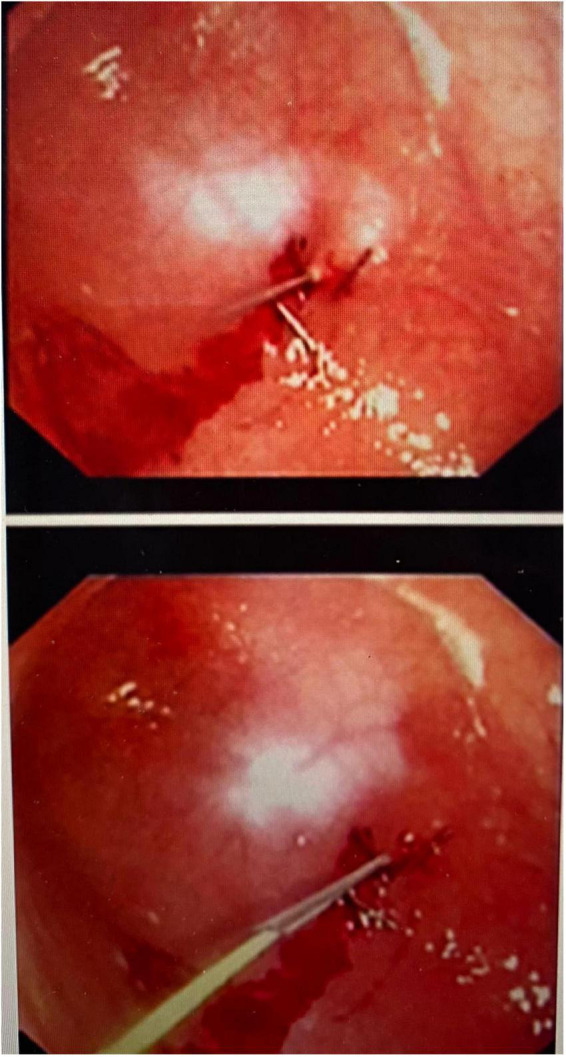
Tuckers placed and guide-wire inserted under endoscopic visualisation.

An initial incision was made at the umbilicus to place a 5-mm optical trocar for the laparoscope. Pneumoperitoneum was achieved at a pressure of 8–12 mmHg. In few cases where adhesions secondary to previous abdominal surgeries such as necrotizing enterocolitis, bowel surgery, diaphragmatic hernia repair or peritoneal catheter insertion, additional ports were placed for better visualization. Once the stomach was identified, an upper endoscopy was performed using a flexible gastroscope. The appropriate site for the gastrostomy insertion was chosen by direct visualization and external finger indentation after insufflating the stomach. The stomach was routinely fixed to the abdominal wall using trans-abdominal trans-gastric tuckers under direct laparoscopic and endoscopic visualization (Figures 1, 2). After fixing the stomach to the abdominal wall, a balloon-type gastrostomy tube was inserted in the gastric lumen with the help of a guidewire (Figure 2) through modified Seldinger technique using a gastrostomy introduction kit. The gastrostomy tube used was either 12 or 14 French depending on patient size. The gastrostomy balloon was finally inflated, and the gastrostomy tube was fixed to the skin at an appropriate length.

### Laparoscopic-assisted gastrostomy

In patients who underwent LAG, the first camera port was inserted by open technique and pneumoperitoneum is sustained at 8–12 mm Hg, with a tendency to use less pressure in smaller children and patients with selected cardiac complications. A 5-mm 30° laparoscope was placed through the umbilical port, and quick abdominal inspection was performed before choosing the appropriate site for gastrostomy tube insertion to avoid creating excessive tension on the gastric wall. With the help of a 3-mm grasping clamp inserted through the planned gastrostomy site, the stomach was grasped along the greater curvature and fixed to the abdominal wall. A guidewire was introduced into the stomach through an 18-G cannula; the passage was subsequently dilated by means of fascial dilators of increasing caliber until a caliber that was wider than the gastrostomy tube to be inserted. The gastrostomy was inserted into the gastric cavity through the push technique, and the balloon was subsequently filled with saline solution. The correct positioning of the gastrostomy was checked by infusing and aspirating methylene blue saline solution.

In both techniques, patients received prophylactic intravenous co-amoxiclav in theater at time of gastrostomy insertion and remained on antibiotics for 24 h. The patients are started on oral rehydration solution on the day following the gastrostomy insertion for 2 h, if the patient tolerated the oral rehydration solution, they will then be started on milk feeds. The patient is generally put on the same milk feed that he was taking prior to gastrostomy insertion. A feeding plan to achieve the target feeding goal is made by a pediatric dietician. Over time we have shifted from next day feeding to feeding on same day the gastrostomy is inserted. The T fastener would normal dissolve spontaneously within a month of insertion. However, if the patient remains hospitalized for more than 5 days, we would cut the tuckers.

Replacement of the gastrostomy tube into a button device occurred 6 weeks after primary insertion.

## Results

In total, 181 children fulfilled the inclusion criteria and underwent either LAG or LAPEG procedure. In total, 92/181 (51%) underwent LAG and 89/181 (49%) underwent LAPEG. The majority of gastrostomies in our series were inserted for patients with neurological disorders 45% such as cerebral palsy, spinal muscular atrophy, hydrocephalus, and lissencephaly. In total, 15% of patients had complex congenital heart disease and some had congenital heart disease as part of a syndrome such as Down’s syndrome, Noonan syndrome, and Di George syndrome. Other less common indications included; 10% had GT insertion following abdominal bowel surgery for necrotizing enterocolitis, bowel atresia, and diaphragmatic hernia, 10% had syndromes with or without swallowing impairment, 8% had metabolic disorders such as glycogen storage disease and urea cycle defects, 4% had chronic lung disease, 4% had renal disorders, and 3% had immune deficiency with associated failure to thrive.

There were no statistically significant differences in patient demographics and patient characteristics between the two groups (Table 1). The youngest patient who underwent LAG was 1 month old, compared to the LAPEG group, where the youngest patient was 3 months old. Most patients (148/181) had an ASA score of 3, 9 patients had a score of 2, 23 patients had a score of 4, and only one patient had a score of 5. In total, 34/92 patient who underwent LAG had previous abdominal surgery such as necrotizing enterocolitis, nephrectomy, peritoneal catheter insertion, ventriculoperitoneal catheter insertion, perforated appendix, midgut volvulus, intestinal atresia, intestinal perforation, diaphragmatic hernia, and exomphalos repair. In total, 29/89 patients who underwent LAPEG had previous abdominal surgery for necrotizing enterocolitis, ventriculoperitoneal shunt insertion, peritoneal catheter insertion, nephrectomy, diaphragmatic hernia, adhesiolysis and fenestration of peritoneal cyst, omphalocele repair, omentectomy, and ligation of patent processes vaginalis. The procedures were elective (outpatients attending for gastrostomy insertion) in 107 patients, including 54 (59%) in the LAG group and 53 (60%) in the LAPEG group. The rest of the patients were in hospital patients requiring prolonged hospital stay for complex and multiple medical disorders and required gastrostomy insertion as part of their overall medical care.

**TABLE 1:** Patient demographics and characteristics.

	LAG (*n* = 92)	LAPEG (*n* = 89)	*P*-Value
Female	43 (46.7)	40 (45)	1.0
Mean age (years)	2.8 (0.08–16)	2.5 (0.25–14)	0.1
Mean weight (kg)	10.3 (2.6–48.4)	10.6 (2.8–33.9)	0.4
Mean American Society of Anesthesiology (ASA) score	3	3	1.0
Previous abdominal surgery	34 (37%)	29 (33%)	0.64
Cardiac disease	19 (21%)	19 (21%)	1.0
Elective cases	54 (59%)	53 (60%)	0.65
PICU patients prior to surgery	37 (40%)	30 (34%)	0.44

Both LAG and LAPEG were performed successfully for patients with no significant complications and no perioperative mortality (Table 2). However, due to poor surgical visibility and limited working space, one patient in each procedure group was converted to open gastrostomy. Only one patient in the LAG group developed a minor serosal injury of the stomach which did not require any surgical intervention. There were no intraperitoneal leaks in either group.

**TABLE 2:** Surgical outcomes and complications.

	LAG (*n* = 92)	LAPEG (*n* = 89)	*P*-Value
Successful completion	91 (99%)	88 (99%)	>0.99
Conversion	1 (1%)	1 (1%)	0.74
Adjacent bowel/organ injury	1 (1%)	0 (0%)	0.51
Intraperitoneal leak	0 (0%)	0 (0%)	
Early tube dislodgement	8 (9%)	7 (6%)	0.53
Repeated early intervention under general anesthesia	1 (1%)	2 (2%)	0.49
Wound infection	13 (13%)	11 (12%)	0.8
Total	29 (32%)	23 (26%)	0.14

Review of the postoperative complications revealed no difference in early tube dislodgement in either group (*p* = 0.53). A total of 15 patients had early tube dislodgement, the earliest tube dislodgement occurred 15 and 10 days following LAG and LAPEG placement, respectively. Only one patient from the LAG and two patients from the LAPEG tube required a second operation under general anesthesia for tube replacement within 15 days of the initial gastrostomy tube insertion. The remaining patients had their gastrostomy tubes replaced successfully at bedside, with no need for additional surgical intervention. In total, 24 (13 LAG and 11 LAPEG) patients had surgical site infection requiring treatment with oral antibiotics. Surgical site infection was identified as erythema, induration, exudate, or purulent secretion at the surgical gastrostomy site within 30 days of the procedure (6). A wound culture was obtained any time considered necessary and treatment with antibiotics was prescribed when considered indicated, which was at the surgeon or gastroenterologists’ discretion. Empiric oral co-amoxiclav was started until the swab result with antibiotic sensitivities were obtained. Antibiotics were changed if the surgical site infection was caused by an organism that is not sensitive to co-amoxiclav. No patient with stoma infection required hospital admission or intravenous antibiotics, there was no deep-seated infection and all patient improved with oral antibiotics. Although there was a trend toward an increased rate of overall surgical complication rate in the LAG group (32%) compared to LAPEG (26%), this result did not achieve statistical significance (*p* = 0.14).

Excluding patients who had a concurrent other surgical procedure (32 LAGs and 40 LAPEGs), the median total operating time was 38 (range 18–66) min in the LAG group and 27 (range 21–74) min in the LAPEG group (*p* < 0.001) (Table 3). The median postoperative hospital stay was 6 days (range 2–20) in the LAG group and 5 days (range 2–16) in the LAPEG group. There was no difference in the LOS between both the groups (*p* = 0.096). Only elective cases were considered while calculating the median length of hospital stay to remove any possible confounders. A total of 74 (41%) patients were transferred to rehabilitation centers for long-term care due to the nature of their underlying comorbidities.

**TABLE 3:** Comparison of median length of stay and operative time.

	LAG	LAPEG	*P*-Value
Median length of hospital stay (days)	6	5	0.096
Median surgery duration (minutes)	38	27	<0.001
LAG/LAPEG performed with another procedure	32 (35%)	40 (45%)	0.36

## Discussion

Gastrostomy tube insertion is a well-established procedure for pediatric patients which can be placed using various techniques. Gastrostomy insertion is indicated when patients have insufficient nutritional or medical intake for a period of >2–3 weeks, or when the need for additional enteral feeding (e.g., through a nasogastric tube) is expected to exceed >3 months (6). Our patients had a variety of medical diseases requiring prolonged enteral tube feeding or specialized feeds for metabolic disorders. The choice of insertion method should be decided after careful consideration of each patient’s clinical scenario, available equipment and local clinicians’ expertise (7). It is important to select the procedure that is associated with the least complications and best outcome for each patient. The two gastrostomy insertion techniques compared in our study are similar with addition of endoscopy to the LAG procedure. In some patients with distorted anatomy traditional PEG insertion can be impossible, these patients will benefit from LAG or LAPEG. Hermanowicz et al. reported twelve patients with cerebral palsy, spastic quadriparesis, severe kyphoscoliosis, and interposed organs who required EN. For these patients PEG placement was deemed impossible. In all patients LAPEG was performed with no complications in the perioperative period (8).

Gastrostomy insertion is associated with a number of complications which can be divided into major and minor complications (9). Major complications include failure of the procedure with the need for conversion to another technique, gastrostomy peritoneal leak causing peritonitis, tube dislodgement, adjacent bowel injury, and gastrocolocutaneous fistula formation. Laparoscopic technique have been demonstrated to be safer than percutaneous endoscopic technique because laparoscopy allows for direct visualization of the peritoneal cavity thereby enabling early detection and avoidance of inadvertent complications of viscus injury during gastropexy and fixation of the stomach to the anterior abdominal wall (10, 11). This apparent advantage thereby minimizes the risks of the serious intra and postoperative complications that require re-operation. Therefore, it is expected that the addition of endoscopy in our study will not demonstrate significant reduction in operative and postoperative surgical complications in the LAPEG group. A meta-analysis reviewing major complications in children after LAG and PEG insertion demonstrated the LAG technique to be associated with 1% compared to 5.4% in the PEG technique. The risk of major complications was higher in PEG than in LAG 3.86 (95% confidence interval 1.9–7.81; *p* < 0.0002) (9). Our study results demonstrated similar findings of low major complication rate in both laparoscopic procedure; LAG and LAPEG. It has been mentioned in previous studies that patients with higher BMI may not be good candidates for LAG procedure because of the technical difficulty in fixation of the stomach through the thicker abdominal wall and possible excessive traction which may potentially result in unrecognized gastric injury (12). In addition, in children with foregut dysmotility and a relatively immobile stomach, attaching the insufflated stomach to the abdominal wall may be challenging (13). We did not specifically review the association between obesity, increased BMI and foregut dysmotility and difficulty in gastrostomy insertion in our group. These unique patient groups may need to be evaluated separately in future studies.

We observe a significantly reduced median operative time for LAPEG compared to LAG by 11 min (*p*-value < 0.001). This can be explained by the fact that endoscopy provided additional visibility of the stomach, allowing for a more confident approach and hence a faster LAPEG procedure. In addition, omitting the step of methylene blue injection to confirm tube placement in LAG may have also contributed to the reduced operative time in the LAPEG group. The median time for LAPEG insertion in our cohort was 27 min, which is similar to previous published data of 20 min (14, 15). However, the median time for LAG in our study is 38 min compared to 75 min as reported by Wragg et al. (16). This added operative time benefit needs to be carefully considered in busy and high-volume centers with prolonged patient waiting time for gastrostomy insertion.

Whilst the median length of hospital stay was shorter by 1 day for patients undergoing LAPEG compared to LAG, this difference was not statistically significant. Some patients in our cohort who had cardiac disease and complex respiratory status require to be transferred to pediatric intensive care following gastrostomy insertion for stabilization for 1–2 days prior to transfer to the ward. We believe that the late introduction of feeds in our patients and the need for stabilization of some patients in pediatric intensive care prior to discharge may explain the prolonged overall hospital stay in both groups. We have recently adopted an enhanced early gastrostomy feeding protocol which will further shorten our hospital post-operative LOS. The cost implication related to increased length of procedure time and increased hospital stay in LAG still needs to be assessed against the need for two clinicians, a surgeon and endoscopist to perform the LAPEG procedure. However, good coordination and harmony between the surgeon and endoscopist is key to achieving a shortened operative time and smooth procedure.

The authors recognize a few limitations to this study. Firstly, this is a retrospective study and as such may be limited by inaccurate documentation. Secondly, since the patients were followed up to 1 year, any major complication beyond the follow-up year had not been accounted for. However, most clinically relevant procedure-related complications associated with gastrostomy insertions are usually identified within a year of initial procedure completion. Thirdly, non-randomization of patients to LAG or LAPEG may have influence the results. Finally, although this study represents the largest review of LAPEG and LAG in children, the authors appreciate that this is still a relatively small sample size which may limit the power of conclusions drawn from this study.

We conclude that this is the first study comparing LAPEG to LAG placement in children. Although, both LAG and LAPEG are safe without significant reported complications, the addition of gastroscopy to the laparoscopic technique in LAPEG allowed for better visualization and eventually a significantly reduced operative time. A larger multi-center randomized controlled trial is required to provide a more comprehensive comparison of LAPEG and LAG in children.

## Data availability statement

The original contributions presented in this study are included in the article/supplementary material, further inquiries can be directed to the corresponding author.

## Ethics statement

The studies involving human participants were reviewed and approved by the Institutional Review Board for Research and Ethics Committee, Sheikh Khalifa Medical City, Abu Dhabi, United Arab Emirates. Written informed consent to participate in this study was provided by the participants’ or their legal guardian/next of kin.

## Author contributions

RB and SA contributed to the conception and design of the work, analysis, interpretation of data, drafting, writing up the work, and revisiting it critically. AS contributed to the design of the work, acquisition, analysis, interpretation of data, and revisiting it critically. AA and MM contributed to the design of the work, interpretation of data, and revisiting it critically. DR and MH contributed to the design of the work, analysis, interpretation of data, and revisiting it critically. All authors contributed to the article and approved the submitted version.
